# Corneal Backscatter Analysis by In Vivo Confocal Microscopy: Fellow Eye Comparison of Small Incision Lenticule Extraction and Femtosecond Laser-Assisted LASIK

**DOI:** 10.1155/2014/265012

**Published:** 2014-03-05

**Authors:** Alper Agca, Engin Bilge Ozgurhan, Yusuf Yildirim, Kadir Ilker Cankaya, Nimet Burcu Guleryuz, Zeynep Alkin, Abdullah Ozkaya, Ahmet Demirok, Omer Faruk Yilmaz

**Affiliations:** ^1^Beyoglu Eye Research and Training Hospital, 34421 Istanbul, Turkey; ^2^Department of Ophthalmology, Istanbul Medeniyet University, 34730 Istanbul, Turkey

## Abstract

*Purpose*. To evaluate and compare corneal backscatter from anterior stroma between small incision lenticule extraction (SMILE) and femtosecond laser-assisted LASIK (femto-LASIK). *Methods*. A cohort of 60 eyes of 30 patients was randomized to receive SMILE in one eye and femto-LASIK in the fellow eye. In vivo confocal microscopy was performed at 1 week and 1, 3, and 6 months after surgery. The main outcome measurements were maximum backscattered intensity and the depth from which it was measured, the backscattered light intensity 30 **μ**m below Bowman's membrane at the flap interface and 150 **μ**m below the superficial epithelium, and the number of refractive particles at the flap interface. *Results*. The mean backscattered light intensity (LI) at all measured depths and the maximum backscattered LI were higher in the SMILE group than the femto-LASIK group at all postoperative visits. LI differences at 1 week and 1- and 3-month visits were statistically significant (*P* < 0,05). LI differences at 6 months were not statistically significant. There was no difference in the number of refractive particles at the flap interface between the groups at any visit. *Conclusions*. SMILE results in increased backscattered LI in the anterior stroma when compared with femto-LASIK were evaluated.

## 1. Introduction

SMILE is a new method for the refractive correction of myopia and myopic astigmatism. In this procedure, an intrastromal lenticule is created between two photodisruption planes and mechanically removed via a 3-4 mm arcuate side cut [1, 2].

The efficacy and safety of SMILE are comparable to those of LASIK. However, the visual recovery after lenticule extraction in SMILE is slower than after LASIK [3, 4]. As the procedure is relatively new, the corneal wound healing response is not well documented, and the reason for delayed recovery is not clear.

A transiently enhanced visibility of the interface in some lenticule extraction eyes during the first postoperative week has been reported, and it is speculated that this type of interface scatter after cutting of the intrastromal lenticule is to blame for the slightly reduced visual acuity in the early postoperative period when compared with LASIK [1, 3, 5]. However, an objective grading of haze in the SMILE group or a comparison of interface haze with the LASIK group has not been performed.

In vivo confocal microscopy (IVCM) can be used to measure endothelial cell density to analyze corneal and intracorneal thicknesses and to assess cellular morphology and histopathologic changes. Another important feature of IVCM is the ability to objectively quantify corneal backscatter, which is used to define stromal reaction, keratocyte activation, and objective haze grading in refractive and lamellar corneal surgery [6–10].

In this study we used corneal backscatter analysis, backscattered light intensity (LI) depth graphics (Z-scan), and subjective evaluation of IVCM images to detect differences between fellow eyes that underwent either SMILE or femto-LASIK.

## 2. Patients and Methods

This prospective study was approved by the ethics committee of the Beyoglu Eye Research and Training Hospital and adheres to the principles of the Declaration of Helsinki. All patients provided informed consent. The study enrolled patients older than 18 years old with myopia or myopic astigmatism with a spherical equivalent refraction of <10 D, mesopic (4 lux), pupil size ≤6.5 mm, and calculated residual stromal bed thickness of >300 *μ*m. Other inclusion criteria were best corrected visual acuity of at least 20/25 in both eyes, no ocular disease other than the refractive error, a normal topographic pattern and regular retinoscopic reflex, corneal pachymetry of >500 *μ*m at the thinnest point, and stable refraction for at least 2 years.

### 2.1. Preoperative and Postoperative Examinations

All patients underwent the standard preoperative refractive surgery procedures of the clinic. All visual acuity measurements were completed using an illuminated ETDRS chart (Optec 3500 Vision Tester, Stereo Optical Co., USA). Objective cycloplegic refraction was performed with an autorefractometer (KR-1 Auto Kerato-Refractometer, Topcon, Japan) and retinoscopy in all patients. Corneal topography, dynamic infrared pupillography, ocular wavefront analysis, and corneal wavefront analysis were performed with a Sirius corneal topography and abberometry system (Costruzioni Strumenti Oftalmici, Italy). Horizontal corneal diameter was measured with an IOLMaster 500 (Carl Zeiss Meditec, Germany). Intraocular pressure was measured with a Goldmann applanation tonometer. All patients underwent a detailed anterior and posterior segment examination using a slit lamp. Optical coherence tomography was performed at the 1-month followup to evaluate flap and cap thicknesses.

### 2.2. Patient Randomization

One eye of each patient was assigned to the SMILE group and the fellow eye to the femto-LASIK group using a random number table. The random numbers were placed in sealed envelopes that were shuffled and then sequentially numbered. The surgeon opened the next available envelope before the surgery. If the random number in the envelope was odd, then the right eye was allocated to the SMILE group, and if the number was even, the left eye was allocated to the SMILE group.

### 2.3. Surgical Technique

The same surgeon (A. Demriok) performed all eye surgeries in the study. For each patient, surgery was performed on both eyes on the same day. The eye in the SMILE group was treated first. The flap of the fellow eye was created before transporting the patient to the excimer laser.

### 2.4. SMILE

A VisuMax (Carl Zeiss Meditec, Germany) femtosecond laser platform was used for all eye surgeries. The same parameters were used in all cases. The spot distance was 3 *μ*m for lamellar cuts and 2 *μ*m for side cuts. The spot energy was set to 140 nJ. The minimum lenticule side cut thickness was set to 15 *μ*m. The lenticule side cut angle was 120°, and the optical zone was 6.5 mm. The optical zone diameter was equal to the lenticule diameter in patients with purely spherical refractive error. However, if the patient had astigmatism, the software added a transition zone to convert the oval lenticule into a circle. As a result, the lenticule diameter was 6.5-6.6 mm depending on the presence or absence of astigmatism. The cap diameter was 7.5 mm with a 50° superior side cut and a side cut angle of 90°. A small-sized (Size S) patient interface was used in all patients. When the lenticule and side cut had been created, the surgeon positioned the eye under the operating microscope of the laser platform using the joystick. Under the operating microscope, a blunt spatula was inserted into the anterior lamellar photodisruption plane to perform dissection of any remaining attachments. The same maneuver was performed in the posterior lamellar photodisruption plane. After the lenticule was completely dissected from the overlying and underlying stroma, it was extracted through the side cut with forceps. An antibiotic drop was applied at the end of the operation.

### 2.5. Femto-LASIK

After one drop of topical anesthetic was applied to both eyes and sterile draping was placed, an eyelid speculum was inserted. Flaps were created by a VisuMax femtosecond laser platform (Carl Zeiss Meditec, Germany). Spot energy was set to 140 nJ. Spot distance was 3 *μ*m for the lamellar flap cut and 2 *μ*m for the flap side cut. The flap side cut was 90°, and the flap diameter was set to 8.5 mm in all patients. A medium-sized (Size M) patient interface was used in all patients. After the flap was created, the patient was transported to a Schwind Amaris 750S (SCHWIND eye-tech solutions, Germany) excimer laser platform. The flap was lifted with a blunt spatula (Katena, USA), and excimer laser photoablation was performed. The residual stromal bed was washed with balanced salt solution, and the flap was repositioned. An antibiotic drop was applied at the end of the operation.

### 2.6. Confocal Microscopy

Confocal microscopy (Confoscan 4, Nidek, Italy) was performed at week 1 and months 1, 3, and 6. The same experienced technician (A. Agca), who had no involvement in the operative procedures, performed all the examinations. The device was equipped with a standard ×40 water-immersion front lens and a Z-ring. One drop of Viscotears (Novartis Pharma AG, Basel, Switzerland) was applied as an immersion substance between the ×40 objective lens and the Z-ring before each examination.

A sterile small wire-lid speculum was inserted into the patient's eye after topical anesthesia to improve examination quality. The technician asked the patient to look at the internal fixation light and manually centered on the endothelium before activating the microscope's autoalignment. The device was used in full-thickness mode. The images were immediately reviewed by the technician and the first author. The procedure was repeated if there were any concerns about the quality or centration of the images or the stability of centration and quality throughout the image acquisition period. The backscattered LI measurements were standardized by using Amco Clear turbidity standard (GFS Chemicals Inc., USA) and presented in scatter units as previously described [11]. Turbidity is measured in nephelometric turbidity units (NTU). The relation between turbidity and image intensity can be used to express image intensity in SUs, provided that 1 SU is equal to the image intensity measured in a 1-NTU suspension [10–12]. AMCO clear is a commercially available calibration standard for measuring turbidity. In our study, we examined AC-4000 (AMCO Clear in a maximum concentration equivalent to 4000 NTU) through a transparent rectangular 10 mm sample cell (LPZ045; Hach Lange, Tiel, The Netherlands) and then, after gradual dilution, AC-2000, AC-1000, and AC-500. Hillenaar et al. showed that the relation between image intensity and turbidity depends on the imaging depth and that a linear relationship with turbidity only exists at a depth image intensity of 200 *μ*m [11]. Thus, the image intensities at a depth of 200 *μ*m were plotted against the turbidity values, and the linear function defining the relation between them was used as the standardization function. This calculation was performed only once (at the beginning of the study) to define a standardization function that could be used to convert the ConfoScan 4 raw image intensity data to SU. To ensure standardization of backscatter measurements over a long time period, a polymethylmethacrylate slab (PMMA, Opal 040 Perspex GS; Lucite International Ltd., UK) was used every week [11, 12].

During pre- and postoperative visits, backscattered LI was measured at the flap/cap interface, at the flap/cap-stromal bed interface, and at the stromal bed. In postoperative visits, the peak backscattered LI in the anterior stroma and its corresponding corneal depth were also recorded. The border of the anterior stroma was determined as described by Hillenaar et al. [12]. In addition, the reflective particles at the flap/cap interface were counted manually.

### 2.7. Flap and Cap Tissues

A backscattered LI value was recorded 30 *μ*m below Bowman's membrane as this location approximately represents the middle portion of the flap or cap stroma in a 120 *μ*m flap or cap.

### 2.8. Flap/Cap-Stromal Bed Interface

The interface was identified by the presence of refractive particles. The image with the clearest view of the refractive particles was selected to represent the flap/cap-stromal bed interface. The intended flap and cap thicknesses were 120 *μ*m in all eyes. Accordingly, if it was difficult to choose between two images, the one that was nearest to 120 *μ*m from the last focused corneal epithelium image was chosen.

### 2.9. Stromal Bed

Corneal stroma 150 *μ*m below the superficial epithelium was chosen to represent the stromal bed, just below the stromal interface.

### 2.10. Statistical Analysis

The distributions of variables were determined with Kolmogorov-Smirnov tests. The mean, standard deviation, and frequency were used in the statistical analysis. The preoperative data were compared using Student's *t*-test (Table 1). The preoperative and postoperative data were analyzed using two-way repeated measure ANOVA with post hoc multiple comparisons. IBM SPSS 20.0 (IBM Corporation, USA) software was used to analyze the data.

## 3. Results

Sixty eyes of 30 patients were included in the study. The mean age of the patients was 27 ± 5 years. Fourteen patients (47%) were male and 16 patients (53%) were female. There were no statistically significant differences in preoperative characteristics of the eyes in the SMILE and femto-LASIK groups (Table 1, *t*-test, *P* > 0.05). The mean flap/cap thicknesses at month 1 visit were 124 ± 9.51 *μ*m in the femto-LASIK group and 122 ± 10.16 *μ*m (*P* > 0.05) in the SMILE group. The mean thicknesses of the removed tissue (the maximum thickness of the extracted lenticule in the SMILE group and the maximum ablation depth in the femto-LASIK group) were 80 ± 28.8 *μ*m and 74 ± 27.1 *μ*m in the SMILE and femto-LASIK groups, respectively (*P* > 0.05). No peri- or postoperative complications occurred.

The maximum value of the backscattered LI increased in both groups postoperatively (Table 2). The increase was higher in the SMILE group, and the difference between the groups was statistically significant at week 1 (*P* < 0.001), month 1 (*P* = 0.009), and month 3 visits (*P* = 0.008). The difference in the amount of increase was not significant at month 6 visit (*P* = 0.245).

Table 3 shows the depth of the maximum backscattered LI throughout the follow-up period. At the postoperative week 1 and month 1 visits, there was no statistically significant difference between the SMILE and femto-LASIK groups in terms of the maximum backscattered LI depths. However, at months 3 and 6 visits, the corneal depth at the maximum backscattered LI was more anterior in the SMILE group (month 3, *P* = 0.02; month 6, *P* = 0.01) than in the femto-LASIK group.

Table 4 shows the backscattered LI 30 *μ*m below Bowman's membrane (cap and flap tissues in the SMILE and femto-LASIK groups, resp.). LI in the flap or cap stroma increased postoperatively in both groups; however, the increase was higher in the SMILE group, and the difference was statistically significant at week 1 (*P* < 0.001), month 1 (*P* = 0.007), and month 3 (*P* = 0.003) visits. Although the mean value was still higher in the SMILE group at the 6-month visit, the difference was not statistically significant (*P* = 0.065).

A similar pattern was observed for the backscattered LI values at the intended flap (Table 5). There were postoperative increases in backscattered LI values in both groups. The increase was higher in the SMILE group and the difference was statistically significant at week 1 (*P* < 0.001), month 1 (*P* = 0.012), and month 3 visits (*P* = 0.006) but not at month 6 visit (*P* = 0.051).

Table 6 shows backscattered LI values at the corneal stroma below the interface level (150 *μ*m). Backscattered LI at this depth increased in both groups postoperatively and the increase was higher in the SMILE group. However, the difference was only statistically significant at week 1 (*P* < 0.001) and month 1 visits (*P* = 0.012).

**Figure 1:**
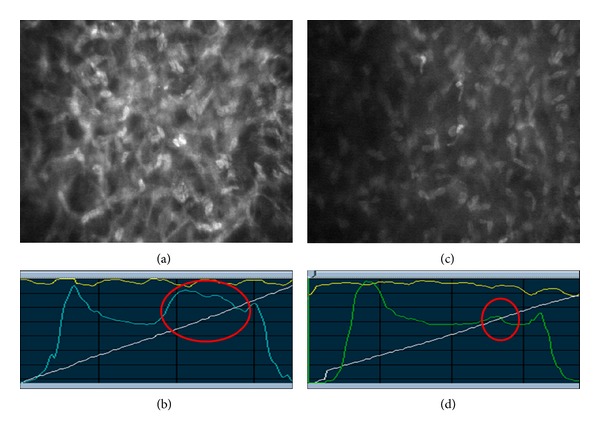
Fellow eyes of the same patient 1 week after surgery. (a) Activated keratocytes (at 150 *μ*m depth) organized as a network after SMILE surgery. (b) Anterior stroma of the same eye shows dramatically increased corneal backscatter (circle). (c) Keratocytes (at 150 *μ*m depth) in the fellow eye that underwent femto-LASIK surgery. (d) After femto-LASIK surgery, there is a limited increase in corneal backscatter compared with SMILE (circle).

Subjective evaluation of backscattered LI versus depth graphics (Z-scan) revealed increased corneal backscatter in a wider area in SMILE eyes. A network of activated keratocytes and increased reflectivity from the extracellular matrix was easily identified in SMILE eyes, and in some patients, this area occupied the anterior one-third of the corneal stroma in the first postoperative week (Figure 1(b)). In contrast, increased backscatter in fellow eyes that underwent femto-LASIK was limited to close proximity of the flap interface (Figure 1(d)).

Table 7 shows the number of reflective particles. The difference was not statistically significant at any postoperative visit.

## 4. Discussion

When reporting on corneal backscatter, image intensity in gray levels should be adjusted to absolute scatter units [11, 12]. McLaren et al. [10] reported mean values for the mean image intensity in each five percentiles of depth. Hillenaar et al. [12] developed an algorithm that semiautomatically calculates the mean corneal backscatter of the whole stroma as well as the mean backscatter in the anterior, middle, and posterior third of the stroma. Although we standardized our measurements according to Hillenaar et al., we were not interested in the mean values for thicker corneal sections obtained by averaging values from different depths; instead, we were interested in detecting even a localized small difference between the groups that may not have affected the mean backscattered LI in thicker corneal sections. Thus, we measured backscattered LI in three specific locations around the flap/cap interface where we most expected to find a difference; in addition, we measured the maximum LI in the anterior stroma and its location. There have been no previous studies comparing IVCM in SMILE and femto-LASIK eyes, and we were uncertain which postoperative period we should concentrate on in order to detect any possible differences. As a result, we performed IVCM as early as 1 week after the surgery and performed it quite frequently (four times in 6 months). Only 0.14% of the corneal surface is imaged in IVCM, and a tracking system is not present in confocal microscopy devices [13]. As a result, positional repeatability is low. However, centralization of the scan on the corneal apex can be verified by obtaining a uniform image of the corneal endothelium. In our opinion, corneal haze in this group of patients could be monitored with sufficient repeatability only if the central cornea was imaged in all cases. Accordingly, we took maximum care to acquire perfectly centralized measurements in every scan. Further positional repeatability is practically impossible with current confocal microscopy technology.

In some studies on lenticule extraction, the very slight clinical haze at the interface is not even mentioned [4, 14]. The status of the interface may not have been recorded by some authors because it may not have been considered to be related to visual symptoms; others may have not reported it, as the design of their studies lacked an objective instrument to detect and measure the interface haze. In the few studies where it was reported, there is no detailed information or measurement of interface haze after lenticule extraction and there is no comparison of it with a LASIK interface. Vestergaard et al. compared 40 femtosecond lenticule extraction (FLEX) eyes with 41 femtosecond laser eyes [3]. The data on the femtosecond laser eyes were retrospectively collected. They reported that slit-lamp examination showed a transiently enhanced visibility of the interface in some FLEX eyes during the first postoperative week; however, they provided no further data (e.g., number of eyes with enhanced visibility, duration of haze). They stated that the delayed recovery may have been related to increased transient haze. Blum et al. [1] and Sekundo et al. [15] also reported a transient haze in some FLEX eyes; however, they did not give a measure of it, nor did they compare it with femto-LASIK.

In this study typical differences were observed in the objective corneal backscatter analysis of corneas between SMILE and femto-LASIK eyes. Eyes that underwent SMILE and femto-LASIK both showed increased postoperative backscattered LI; however, maximum backscattered LI and backscattered LI from three different depths in the anterior stroma were higher in SMILE eyes. In fact, the difference in the early postoperative period was so dramatic that, even without measuring the actual intensity levels or performing a statistical analysis, we could easily identify that there was an obvious difference between the eyes (Figures 1(a) and 1(c)). Subjective evaluation of confocal microscopy images revealed that increased reflectivity in the SMILE eyes was due to the extracellular matrix and activated keratocytes (Figure 1(a)). The difference was not likely to be due to individual immunological differences because SMILE and femto-LASIK were compared on fellow eyes operated on in the same day. In addition, the amount of preoperative refractive error, the thickness of the tissue removed from the stroma, and the depth from which the removal (ablation or extraction depending on the surgical method) took place were similar between the groups, and the patients were given the same postoperative drug regimen. Thus, the groups were perfectly matched, and the only difference was the surgical procedure. We found that the difference between the groups in terms of backscattered LI was the highest at week 1 and reduced gradually thereafter. There was no statistically significant difference between the groups at 6 months postoperatively. Interestingly, the location of the peak backscattered LI was more anterior in the SMILE group at 3 and 6 months postoperatively (Table 3). At month 6 visit, the location of the peak backscattered LI was at, or slightly above, the interface in the SMILE group (106 ± 27 *μ*m) and at the level of, or slightly below, the interface (142 ± 27 *μ*m) in the femto-LASIK group.

The only confocal microscopy study comparing lenticule extraction and femto-LASIK was carried out in a limited number of rabbit eyes by Riau et al. [16]. They performed a semiquantitative analysis of the reflectivity level of the flap interface by measuring the mean gray value of the reflective particles using ImageJ software (National Institutes of Health, USA). The reflectivity level was not standardized in their study. As a result, although it is possible to make a comparison between the two groups in that particular study, the values cannot be compared with any other study group examined with a different confocal microscope. Contrary to our findings, they reported that the relative reflectivity of the flap interface was significantly higher in femto-LASIK eyes. They concluded that refractive lenticule extraction may result in less inflammation and less extracellular matrix deposition compared to LASIK, especially at high refractive correction. Their study included 36 eyes evenly divided between the FLEX and femto-LASIK groups. However, an overall comparison of the two groups was not performed. Instead, each group was divided into three subgroups, with six eyes in each subgroup (subgroups with −3.00, −6.00, and −9.00 D corrections). Each subgroup of the FLEX eyes was compared with its corresponding femto-LASIK subgroup (the difference between the surgical procedures was statistically significant in two of the three subgroups). As a result, the statistics and study conclusions were based on the means of six eyes for each subgroup; results on such a small number of eyes may be misleading. In addition, rabbit eyes may not be comparable to human eyes.

LASIK leads to a variably thick, hypocellular, primitive stromal interface scar [17]. By using confocal microscopy, the interface wound can be identified in 100% of cases because the scar always contains numerous brightly reflective interface particles. These reflective particles were found to consist primarily of organic cellular constituents in histologic studies [17]. Riau et al. reported more intense and abundant reflective particles in those corneas that underwent femto-LASIK compared with those that underwent FLEX. However, the number of these reflective particles did not differ between groups in the present study.

The more superficial lamellar cut made in the SMILE surgery is similar to the flap cut that is created in femto-LASIK. Thus, the difference between the surgical procedures is the result of the addition of a second lamellar cut at a deeper level or the increased number of surgical steps required for blunt separation of the lenticule floor and roof in the SMILE group. In femto-LASIK, only one lamellar cut is performed with a femtosecond laser to create the flap, and the incidence of deep lamellar keratitis was found to be consistently higher after femto-LASIK when compared with LASIK using a mechanical keratome [18–21]. Thus, the application of a femtosecond laser on the corneal stroma may have unique immune consequences. In SMILE, two lamellar cuts are performed, and when the lenticule is extracted, these two surfaces come face to face at the cap-stromal bed interface, where our measurements were recorded. In addition, the total energy applied to the corneal stroma in SMILE is higher than that applied to the stroma in femto-LASIK performed with the same platform. The surgical maneuvers used in the SMILE procedure are far more challenging than those used in FLEX and femto-LASIK procedures, so varying inflammatory responses could equally as well be related to the surgical maneuvers as to the effects of the laser treatment [2]. A study comparing FLEX and SMILE procedures would help to reveal whether increased surgical maneuvers increase postoperative corneal backscatter. Vestergaard et al. reported that refractive predictability and corneal aberrations following FLEX seemed better than or equal to those following femto-LASIK at 3 months, whereas visual recovery after FLEX was slower [3]. R. Shah and S. Shah also reported that although the efficacy and safety of refractive lenticule extraction (FLEX and SMILE) are comparable to those of LASIK, visual recovery is slower, and changing the scanning trajectory of the femtosecond laser had a positive effect on visual outcome [4]. Another possible factor that delays visual recovery may be the different patterns of stromal response to lenticule extraction and femto-LASIK. In fact, it should not be surprising to find different patterns of stromal response to lenticule extraction and femto-LASIK because they are different surgical procedures.

In conclusion, we report for the first time that SMILE eyes have increased corneal backscatter for at least 3 months after surgery at the level where the lenticule is extracted compared with femto-LASIK eyes. This increased corneal backscatter in the early postoperative period does not necessarily result in decreased vision, increased incidence of complications, or increased inflammation, but it obviously reflects a different healing response after SMILE. Whether this difference has any positive or negative clinical consequences is unknown at this time. However it is speculated that suboptimal cutting of the stromal fibers by the femtosecond laser may be the reason for this type of interface scatter, which results in slightly reduced visual acuity in the early postoperative period [3, 5]. If this is correct, incorporating IVCM evaluation of the interface into future studies on laser energy, spot size, and spacing settings may help to further optimize these factors and minimize a delay in recovery.

## Figures and Tables

**Table 1:** Preoperative characteristics.

	SMILE (*n* = 30)	Femto-LASIK (*n* = 30)	*P*
	Mean ± SD	Mean ± SD	
Preoperative spherical equivalent (D)	−4.00 ± 1.63	−3.20 ± 1.58	0.120
Preoperative mean keratometry (Keratometric D)	44.01 ± 1.41	44.06 ± 1.33	0.851
Preoperative thinnest corneal pachymetry (*µ*m)	544 ± 28	543 ± 29	0.954
Preoperative endothelial cell count (cells/mm^2^)	2814 ± 228	2780 ± 221	0.160
Thickness of the removed tissue* (*µ*m)	80 ± 28.8	74 ± 27.1	0.761

*n*: number of eyes; SD: standard deviation; D: diopters; *µ*m: micrometer.

*Intended maximum lenticule thickness in the SMILE group; intended maximum ablation depth in the femto-LASIK group.

**Table 2:** Maximum backscattered light intensity in anterior stroma.

	SMILE (*n* = 30)	Femto-LASIK (*n* = 30)	*P*
	Mean (SU) ± SD	Mean (SU) ± SD	
Preoperative	1147.50 ± 85.77	1138.50 ± 104.04	0.020
Week 1*	1793.70 ± 241.74	1450.80 ± 247.32	
Month 1*	1539.00 ± 272.88	1348.11 ± 199.71	
Month 3*	1390.77 ± 260.46	1226.16 ± 183.24	
Month 6	1242.00 ± 129.33	1232.01 ± 127.44	

*n*: number of patients; SU: scatter units; SD: standard deviation.

*P < 0.05: the difference between the two groups in the change from baseline.

**Table 3:** Maximum backscattered light intensity depth.

	SMILE (*n* = 30)	Femto-LASIK (*n* = 30)	*P*
	Mean (*µ*m) ± SD	Mean (*µ*m) ± SD	
Week 1	125.10 ± 27.50	115.60 ± 19.65	0.180
Month 1	119.42 ± 23.13	129.47 ± 30.19	0.235
Month 3*	108.00 ± 22.49	132.41 ± 28.64	0.02
Month 6*	106.44 ± 27.37	142.11 ± 27.26	0.01

Independent samples *t* test.

*n*: number of eyes; µm: micrometer; SD: standard deviation.

*The difference between the two groups is statistically significant.

**Table 4:** Backscattered light intensity 30 *µ*m below Bowman's membrane.

	SMILE (*n* = 30)	Femto-LASIK (*n* = 30)	*P*
	Mean (SU) ± SD	Mean (SU) ± SD	
Preoperative	1116.45 ± 85.59	1099.35 ± 91.44	0.012
Week 1*	1706.85 ± 238.23	1396.80 ± 236.52	
Month 1*	1464.66 ± 252.36	1299.78 ± 164.70	
Month 3*	1370.61 ± 247.23	1161.00 ± 173.61	
Month 6	1215.99 ± 122.58	1144.98 ± 151.74	

*n*: number of eyes; SU: scatter units; SD: standard deviation.

*P < 0.05: the difference between the two groups in the change from baseline.

**Table 5:** Backscattered light intensity 120 *μ*m below the epithelium.

	SMILE (*n* = 30)	Femto-LASIK (*n* = 30)	*P*
	Mean (SU) ± SD	Mean (SU) ± SD	
Preoperative	1062.45 ± 75.51	1051.20 ± 88.83	0.007
Week 1*	1728.90 ± 238.50	1391.40 ± 238.68	
Month 1*	1491.12 ± 288.36	1296.45 ± 199.35	
Month 3*	1340.46 ± 257.49	1163.16 ± 191.16	
Month 6	1212.03 ± 169.92	1124.01 ± 148.23	

*n*: number of eyes; SU: scatter units; SD: standard deviation.

*P < 0.05: the difference between the two groups in the change from baseline.

**Table 6:** Backscattered light intensity 150 *µ*m below the epithelium.

	SMILE (*n* = 30)	Femto-LASIK (*n* = 30)	*P*
	Mean (SU) ± SD	Mean (SU) ± SD	
Preoperative	1001.25 ± 85.32	995.40 ± 88.74	0.017
Week 1*	1671.75 ± 275.58	1312.65 ± 244.89	
Month 1*	1427.67 ± 289.44	1292.67 ± 227.16	
Month 3	1246.23 ± 207.00	1181.61 ± 198.18	
Month 6	1161.99 ± 167.76	1193.04 ± 163.71	

*n*: number of eyes; SU: scatter units; SD: standard deviation.

*P < 0.05: the difference between the two groups in the amount of change from baseline.

**Table 7:** Number of reflective particles.

	SMILE (*n* = 30)	Femto-LASIK (*n* = 30)	*P *
	Mean (µm) ± SD	Mean (µm) ± SD	
Week 1	352.9 ± 290.3	236.1 ± 167.5	0.136
Month 1	262.1 ± 234.8	190.4 ± 140.1	0.273
Month 3	222.3 ± 197.6	130.3 ± 87.3	0.110
Month 6	152.9 ± 116.4	161.8 ± 91.2	0.761

Independent samples *t*-test.

*n*: number of eyes; µm: micrometer; SD: standard deviation.

## References

[1] Blum M, Kunert K, Schröder M, Sekundo W (2010). Femtosecond lenticule extraction for the correction of myopia: preliminary 6-month results. *Graefe’s Archive for Clinical and Experimental Ophthalmology*.

[2] Sekundo W, Kunert KS, Blum M (2011). Small incision corneal refractive surgery using the small incision lenticule extraction (SMILE) procedure for the correction of myopia and myopic astigmatism: results of a 6 month prospective study. *British Journal of Ophthalmology*.

[3] Vestergaard A, Ivarsen A, Asp S, Hjortdal JØ (2013). Femtosecond (FS) laser vision correction procedure for moderate to high myopia: a prospective study of ReLEx® flex and comparison with a retrospective study of FS-laser in situ keratomileusis. *Acta Ophthalmologica*.

[4] Shah R, Shah S (2011). Effect of scanning patterns on the results of femtosecond laser lenticule extraction refractive surgery. *Journal of Cataract and Refractive Surgery*.

[5] Heichel J, Blum M, Duncker GIW, Sietmann R, Kunert KS (2011). Surface quality of porcine corneal lenticules after femtosecond lenticule extraction. *Ophthalmic Research*.

[6] Møller-Pedersen T, Vogel M, Hong Fang Li HFL, Petroll WM, Cavanagh HD, Jester JV (1997). Quantification of stromal thinning, epithelial thickness, and corneal haze after photorefractive keratectomy using in vivo confocal microscopy. *Ophthalmology*.

[7] McCulley JP, Petroll M (2008). Quantitative assessment of corneal wound healing following intralasik using in vivo confocal microscopy. *Transactions of the American Ophthalmological Society*.

[8] Marchini G, Mastropasqua L, Pedrotti E, Nubile M, Ciancaglini M, Sbabo A (2006). Deep lamellar keratoplasty by intracorneal dissection: a prospective clinical and confocal microscopic study. *Ophthalmology*.

[9] Prasher P, Muftuoglu O, Bowman RW (2009). Tandem scanning confocal microscopy of cornea after descemet stripping automated endothelial keratoplasty. *Eye and Contact Lens*.

[10] McLaren JW, Bourne WM, Patel SV (2010). Standardization of corneal haze measurement in confocal microscopy. *Investigative Ophthalmology and Visual Science*.

[11] Hillenaar T, Sicam VADP, Vermeer KA (2011). Wide-range calibration of corneal backscatter analysis by in vivo confocal microscopy. *Investigative Ophthalmology and Visual Science*.

[12] Hillenaar T, Cals RHH, Eilers PHC, Wubbels RJ, van Cleynenbreugel H, Remeijer L (2011). Normative database for corneal backscatter analysis by in vivo confocal microscopy. *Investigative Ophthalmology & Visual Science*.

[13] Hillenaar T, Weenen C, Wubbels RJ, Remeijer L (2009). Endothelial involvement in herpes simplex virus keratitis: an in vivo confocal microscopy study. *Ophthalmology*.

[14] Shah R, Shah S, Sengupta S (2011). Results of small incision lenticule extraction: all-in-one femtosecond laser refractive surgery. *Journal of Cataract and Refractive Surgery*.

[15] Sekundo W, Kunert K, Russmann C (2008). First efficacy and safety study of femtosecond lenticule extraction for the correction of myopia: six-month results. *Journal of Cataract and Refractive Surgery*.

[16] Riau AK, Angunawela RI, Chaurasia SS, Lee WS, Tan DT, Mehta JS (2011). Early corneal wound healing and inflammatory responses after refractive lenticule extraction (ReLEX). *Investigative Ophthalmology and Visual Science*.

[17] Dawson DG, Holley GP, Geroski DH, Waring GO, Grossniklaus HE, Edelhauser HF (2005). Ex vivo confocal microscopy of human LASIK corneas with histologic and ultrastructural correlation. *Ophthalmology*.

[18] Zhang Z-H, Jin H-Y, Suo Y (2011). Femtosecond laser versus mechanical microkeratome laser in situ keratomileusis for myopia: metaanalysis of randomized controlled trials. *Journal of Cataract and Refractive Surgery*.

[19] Chen S, Feng Y, Stojanovic A, Jankov MR, Wang Q (2012). Intralase femtosecond laser vs mechanical microkeratomes in LASIK for myopia: a systematic review and meta-analysis. *Journal of Refractive Surgery*.

[20] Moshirfar M, Gardiner JP, Schliesser JA (2010). Laser in situ keratomileusis flap complications using mechanical microkeratome versus femtosecond laser: retrospective comparison. *Journal of Cataract and Refractive Surgery*.

[21] Gil-Cazorla R, Teus MA, de Benito-Llopis L, Fuentes I (2008). Incidence of diffuse lamellar keratitis after laser in situ keratomileusis associated with the IntraLase 15 kHz femtosecond laser and Moria M2 microkeratome. *Journal of Cataract and Refractive Surgery*.

